# An early intervention for adolescent depression targeting emotional mental images and memory specificity: a process evaluation

**DOI:** 10.1007/s00787-021-01902-7

**Published:** 2021-11-16

**Authors:** Victoria Pile, Laura K. Schlepper, Jennifer Y. F. Lau, Mary Leamy

**Affiliations:** 1 Department of Psychology, Institute of Psychiatry, Psychology and Neuroscience, King’s College London, De Crespigny Park, London, SE5 8AF UK; 2 Nuffield Trust, 59 New Cavendish Street, London, W1G 7LP UK; 3 Florence Nightingale Faculty of Nursing and Midwifery, King’s College London, London, UK

**Keywords:** Depression, Adolescence, Process evaluation, Mental imagery, Memory specificity

## Abstract

We have evaluated a novel early intervention for adolescent depression (age 16–18) in a feasibility randomised controlled trial. This nested process evaluation aimed to understand how this complex intervention worked. We sought to understand participants’ views and experiences of receiving and interacting with the intervention to evaluate whether the underpinning theoretical basis of the intervention is justified and whether it contributes to valued outcomes for participants. Twelve participants were invited to take part in semi-structured interviews. Framework analysis was employed to identify important aspects of adolescents’ experiences. The active ingredients identified by participants were consistent with and extended our understanding of the theoretical basis of the intervention. Four principle themes were identified: understanding how memory works and being able to remember memories in more detail; processing negative experiences and letting go; imagining positive future events; and understanding and being kinder to myself. The outcomes of the intervention were valued by participants. Six principle themes were identified: improving mood and well-being; reducing impact of negative memories; motivation and goal-directed behaviour; overcoming avoidance and rumination; relationships, communication and being open; and self-understanding and acceptance. A simplified logic model is also proposed to connect the intervention components, active ingredients, and valued outcomes. The findings provide an in-depth understanding of how participants interacted with the intervention and what they derived from it. For example, the findings establish processing negative experiences as a core intervention component, extend it to include letting go of these memories, and highlight that reducing the impact of negative memories is valued by participants. This richer understanding guides further intervention development and future implementation.

## Introduction

Depression most commonly begins in adolescence. Adolescent-onset, rather than adult-onset, is associated with more severe social and psychological outcomes [1, 2]. These long-lasting negative outcomes could be mitigated with effective early interventions in schools [2, 3]. Whilst there have been a number of interventions developed previously, most fail to demonstrate evidence of effectiveness and few are easy to access [4–6]. Moreover, there is a well-recognised problem in retaining treatment effect sizes when scaling up small-scale trials into larger trials and into clinical settings. Giving a voice to young people in designing and implementing new interventions and healthcare systems has long been recognised as a priority [7]. Therefore, it is important that in developing new interventions for this group, a clear understanding of what the active therapeutic ingredients are, how they contribute to the effectiveness of the intervention, and whether the outcomes are valued by participants, is established.

Clinical guidelines recommend a stepped-care approach for identifying and intervening with depression [8]. Schools have been consistently identified as a valuable place to improve identification and to embed early interventions, aiming to increase access and prevent problems deteriorating [3]. Deriving knowledge from cognitive science about the underlying processes that drive and maintain depression offers insights into what could be effective targets for clinical interventions. There is evidence that maladaptive autobiographical memory processes (e.g., memory specificity) and related dysfunctional mental imagery of the past and future are associated with depression in adults [9, 10] and in adolescence [11, 12]. There are a number of possible approaches to translating findings from cognitive science into interventions. One approach is to only target the key cognitive process (e.g., memory specificity) in the intervention to reduce complexity and improve scalability. However, as yet these interventions have mostly only been effective in modifying the targeted mechanism and not in reducing depressive symptoms compared to a control group [13]. Complex interventions, which have several interacting components [14], are more common in clinical practise and perhaps more likely to effectively reduce depression. Therefore, another approach is to develop a complex intervention and then evaluate it using mixed methods, and to identify whether the hypothesised active ingredients are important and whether they translate into outcomes that are valued by participants. One-to-one qualitative interviews were selected to enable participant’s experiences of receiving and interacting with the intervention to be explored sensitively. Framework analysis ensured an in-depth exploration of the data, whilst enabling the research to capture a breadth and diversity of experiences.

We have developed a complex intervention derived from cognitive science yet consistent with current practise. We conducted qualitative interviews in the context of a feasibility randomised controlled trial (RCT) in secondary schools in London [15, 16]. The process evaluation aimed to understand (1) the active ingredients of the intervention which the participants felt contributed to therapeutic change, and (2) what participants felt the outcomes from the intervention were and whether they valued these outcomes. We also propose a framework to understand how these components interact. This will contribute to the development and application of the intervention; enabling us to amplify any benefits and reduce any costs [13, 16]. In addition, the qualitative interviews provided an initial assessment of acceptability and feasibility of the intervention, which is reported in the trial paper.

This psychological intervention for young people aged 16–18 combines techniques of imagery rescripting (IR) for negative events, image generation for positive future events, and memory specificity training to produce a novel early intervention for adolescent depression. The main proposed active ingredients of the intervention were: (1) improved understanding of how memory works and its relationship with mood and behaviour; (2) reducing the impact of negative past imagery using IR [17–19]; (3) improving access to positive future imagery [20]; and (4) improving access to specific memories and linking these to an individual’s values [21, 22]. In addition, non-specific therapist factors (empathy, active listening; therapeutic relationship) are very likely to contribute to change. For further information on the intervention components, please see the RCT protocol and publication [15, 16]. Given that this intervention is highly novel and has a number of hypothesised active ingredients, the qualitative interviews help us to understand whether the theoretical basis of the intervention is justified and whether it contributes to valued outcomes for participants. They provide rich insights into whether our understanding of how the intervention might work is supported by the participant’s views and experiences of the intervention. Our primary research questions were:

1. Which aspects of the intervention do participants think led to therapeutic change for them?
2. In what ways do participants think that the intervention has helped them and are these outcomes valued by participants?

## Methods

### Setting for the study

These qualitative interviews were carried out for the evaluation of the IMAGINE trial, a feasibility randomised controlled trial (RCT) based in secondary schools. The trial was prospectively registered on ISCTRN registry (https://www.isrctn.com/; ISRCTN85369879). Ethical approval was obtained from the obtained from the Psychiatry, Nursing and Midwifery Research Ethics Committee at Kings College London (ref: HR-16/17-3548) and the full trial protocol was published [15]. The inclusion criteria for the trial included being aged 16–18 and scoring above clinical cut-off on the Mood and Feelings questionnaire [23]. Exclusion criteria for the trial included currently receiving another psychological intervention and factors contra-indicating IR (i.e., high levels of current risk).

### Intervention: imagery-based cognitive behavioural intervention (IBCI)

ICBI was delivered individually in schools by a Clinical Psychologist (first author) to young people aged 16–18. It combined (a) imagery rescripting (IR) to reduce the distress associated with a negative image and to build a positive future image; and (b) memory specificity training (MEST) to increase specificity and access to memories. The manualised intervention uses cognitive behavioural procedures (e.g., an agenda and homework) and is accompanied by a workbook. IR for a negative past image consisted of three stages: (1) reliving the memory as themselves; (2) reliving the image as a compassionate other who is able to intervene; (3) reliving image as themselves but with the intervention from the compassionate other. Generating a positive future image similarly involved three steps: (1) imagining a vivid future image; (2) imagining they have achieved this future goal (future me) and speak to their current self (current me) to offer advice, speak about what has been positive and what has been difficult, and what has helped them achieve their goal; (3) begin as their current self and progress through a series of smaller images to their future self. For further details, see [24–26]. All imagery exercises are completed in the first person, present tense, and (when happy to) participants are asked to close their eyes. Participants are asked to generate as much detail as possible and are prompted for additional sensory information as well as for thoughts, feelings, and the meaning of the image to them. Homework tasks are delivered via a mobile phone application, which the participants download onto their phones.

### Recruitment

We interviewed participants who completed ICBI until saturation of the principal themes was achieved, namely 12 participants. Participants all provided written informed consent. Participants who completed the control intervention were not interviewed, similar to other studies [27], given that our primary aim was to understand the active ingredients of ICBI. Participants were approached initially by email, and then, a suitable time to meet was arranged. Interviews were conducted after the follow-up period of the trial (3 months after completing the intervention) to ensure that participating in the interviews did not affect the results of the trial. There were only two participants (of 29) who did not complete ICBI and neither of these participants responded to invitations to participate in an interview. A purposive sample of participants (based on gender, baseline depression severity, change in depression score, and school) was utilised to investigate our research questions across different groups. We sought to interview participants with a range of depression scores both in terms of baseline score and change with intervention; depression score was measured using the Mood and Feelings Questionnaire (MFQ; [23], in which change of ten points is considered clinically meaningful and important [28]).

### Data collection and analysis

We used qualitative semi-structured research interviews which were nested within a RCT. The topic guide was developed using the authors’ clinical and research experience in the fields of depression, psychological interventions, and qualitative methodology. Questions were developed alongside our service-user consultants to improve chances of addressing key priorities and reducing risk of assessor bias. Questions were evaluated by the authors, piloted on adolescents, and consequently revised. The topic guide explored the following: (1) why participants wanted to take part in the trial; (2) what they thought about the intervention; (3) how participants thought the intervention might or might not have helped them; (4) their experience of each session and therapeutic techniques. The topic guide was used flexibly and comprised open questions followed by prompts to gather richer data. Constructive feedback was actively encouraged, for example by introducing the interview as an opportunity to help us understand their experience, adapt, and improve the intervention and using prompts throughout to elicit constructive feedback (e.g., Is there anything that you did not like about the programme that you would want to change?). Participants were reminded about the intervention content by the interviewer using a blank therapy workbook. The therapy workbook includes diagrams and worksheets that the participant completes with the therapist during the intervention. It provides a useful illustrative framework to summarise discussions and acts as a visual reminder. For example, for session 2, there is a formulation diagram detailing how negative images can remain distressing. The trial therapist conducted the interviews. The team decided that this was essential to be able to remind participants of what was discussed and reduce recall bias. The interviews were audio-recorded and lasted between 20 and 59 min ($\overline{x }=$ 33 min).

Data were transcribed verbatim and all identifying information removed and pseudonyms assigned. Framework analysis (which sits within a broadly ‘thematic’ approach) was identified as the most suitable method of data analysis. It allows the combination of a priori issues and emergent data-driven categories to develop the analytic framework (Richie and Spencer 1994). This meant that we could both investigate our predefined research questions and have capacity for new insights to emerge from the data. Our analysis was consistent with a recent project analysing interviews from a similar (larger scale) intervention trial of adolescent depression [29, 30]. The results presented here are a synthesis of the themes identified within the framework analysis.

The analysis was conducted in several stages. First, researchers immersed themselves in the young people’s experiences of the intervention by familiarising themselves with the data through reading the transcripts and data analysis meetings. Second, to organise the data, the coding framework was developed by three researchers (VP, who has a background in clinical psychology, LS who has a background in psychology as well as being a service-user consultant on the trial, and ML who has a background in psychology and extensive experience in qualitative analysis). We then piloted this initial framework on two interviewees to further refine our framework and categories, discussing the development of the coding framework as a team. This provided the opportunity to understand any challenges within our coding process and to ensure that our understanding of the categories was consistent across researchers. It also helped us to refine our framework, so that it was a good fit for the issues within the data. The interviews were each coded using the framework by VP and LS. Throughout, the framework developed in a responsive manner to any emergent data-driven issues from each interview and was systematically refined to ensure that concepts were appropriately identified, agreed, and described. VP and LS met regularly, and ML provided consultation on the continual development of the coding framework. Finally, we began to interpret and summarise the data to identify patterns within it. There were no notable disagreements between researchers regarding the identification and description of concepts within the analysis. Analysis was supported by qualitative analysis computer software NVivo 12 [31].

## Results

Twelve of the twenty-nine participants from the experimental arm of the trial were invited and agreed to take part in the interview (see Table 1). All participants were aged 16–18; the majority were female (*n* = 8); and Bangladeshi was the most common ethnicity (*n* = 5). Three participants did not respond to an invitation to take part in the interview. Findings on participants’ decision to take part in the trial (which may inform feasibility) and acceptability of the intervention and technology can be found in the trial paper. In general, the intervention was highly acceptable, but changes to the daily memory training (homework task) will be necessary in a future trial.

1. **Active therapeutic ingredients**

Four principal themes were identified as summarising the active ingredients of the intervention that the participants felt led to therapeutic change. Please see Table 2 for a summary of the themes linked to the overarching categories of active therapeutic ingredients and valued outcomes.

**Table 1:** Participant pseudonyms, demographics, and depression scores

Pseudonym	Age*	Gender	School	Ethnicity	Depression scores				
					T1	T2	T3	Change T2–T1	Change T3–T1
Eleanor	17.64	Female	5	Black/African/Caribbean/Black British-African	43	19	16	− 24	− 27
Ruby	16.91	Female	5	Black/African/Caribbean/Black British-African	28	7	7	− 21	− 21
Ben	16.86	Male	1	White-British	28	7	4	− 21	− 24
Michael	17.26	Male	3	Black/African/Caribbean/Black British-Caribbean	21	11	17	− 10	− 4
Andy	17.93	Male	3	White-British	23	16	6	− 7	− 17
Tanay	16.34	Male	2	Asian/Asian British-Bangladeshi	33	4	6	− 29	− 27
Naisha	16.57	Female	2	Asian/Asian British-Bangladeshi	21	10	12	− 11	− 9
Suravi	16.84	Female	2	Asian/Asian British-Bangladeshi	37	10	5	− 27	− 32
Mishti	16.52	Female	2	Asian/Asian British-Bangladeshi	35	12	4	− 23	− 31
Prisha	18.93	Female	2	Asian/Asian British-Bangladeshi	20	12	24	− 8	4
Alexandra	16.64	Female	1	Black/African/Caribbean/Black British-African	33	23	23	− 10	− 10
Sara	16.84	Female	1	White-British	42	18	14	− 24	− 28

^*^Age at which participants were randomised in the RCT

**Table 2:** Themes and subthemes for each category

Category	Theme	Subtheme
1. Active therapeutic ingredients	1.1 Understanding how memory works and being able to remember memories in more detail	1.1.1 Thinking about how memory works and how memories impact on me 1.1.2 Improving access to and detail of memories 1.1.3 Increasing awareness of positive experience
	1.2 Processing negative experiences and letting go	1.2.1 Compassionate perspective taking
	1.3 Imaging positive future events	
	1.4 Understanding and being kinder to myself	1.4.1 Expressing emotions and understanding myself 1.4.2 Self-compassion 1.4.3 Values for living (and links with memories)
2. Valued outcomes	2.1 Improving mood and well-being	
	2.2 Reducing impact of negative memories	2.2.1 Overcoming rumination to the memory
	2.3 Motivation and goal-directed behaviour	
	2.4 Overcoming avoidance and rumination	
	2.5 Relationships, communication, and being open	
	2.6 Self-understanding and acceptance	

**1.1 Understanding how memory works and being able to remember memories in more detail** This theme captures the sense that the intervention changed the relationship that the participants had with their memories.

*1.1.1 Thinking about how memory works and how memories impact on me (n* = *10)* Young people described a sense of better understanding how their memory and their mood might interact and that this increased understanding had been helpful in enabling them to make changes and observe the processes in real time.

When I kind of realised that, it [the bad memories] all came at once, it kind of gave me a better understanding of it, a grip of it… understanding my memories in a bit more detail, that obviously helped…getting a bit more knowing about what I need to, how my head was working. (Ben)

Participants acknowledged having more respect for their memories, the importance of understanding the meaning of the memories, and the influence that memories can have on them. They spoke about taking a more active role in creating and remembering events and utilising their memories to modulate their mood. Participants described a sense of relief in speaking about memories. An important shift for young people was to have a sense of agency, ownership, and influence over their memories.

I saw my memories in a different way, I see it in a more cinematic way, I suppose, yeah, like I choose to open them and close them. (Tanay)

*1.1.2 Improving access to and detail of memories (n* = *10)* Participants described that the intervention had enabled them to remember events in much more detail.

It really triggered my imagination to think more deeply about past memories, so if I was to think about one now, I’d think about it in a lot of detail. (Naisha)

Several participants said that they were very surprised by the level of detail they were able to recall. Some were challenged by being asked to retrieve specific memories to cue words; most reported that this was a positive process, but others found it too difficult. Examples were given of when they had actively accessed memories and remembered more detail:

So, I remember them and then I tell someone…then I’ll remember even more, then I'm continuing saying, and then she'll say her memories, yeah, and it helps me remember more. (Tanay)

Remembering positive events in detail appeared to act as a reminder for positive memories and the power that these memories can have on their mood and behaviour. They identified that remembering past positive memories helped them to evaluate their current feelings (e.g., stressed) and make changes to reduce these feelings. Several participants acknowledged that they were often attending to their negative memories in preference to their positive memories:

I was quite focusing on all the negative things and so by shifting to all the positive things that happened it did change my behaviour and like changed my day-to-day life as well by remembering those memories (Mishti)

Participants were able to harness these techniques outside of therapy to improve their mood, giving examples of times when they have been able to recall positive memories, for example to alleviate stress or improve mood.

Now I value them [memories] more, and I treasure the ones that I think are most valuable, but I also think about the ones that are sad, but I think of them in a positive light now…it helps me definitely, it definitely changes my mood, helps me think of even more positive… (Naisha)

*1.1.3 Increasing awareness of positive experience (n* = *4) *Some participants described increased optimism and being more aware of positive events as they happen*.* This increased awareness had made them more positive about themselves and that they were able to derive positive self-attributions from these events.

I identify like this is really good and I should be happy or proud that I’ve achieved or done this kind of thing...that’s put me in a better mood. (Alexandra)

**1.2 Processing negative experiences and letting go** Participants (*n* = 11) spoke about the importance of IR for changing their relationship with negative memories and processing negative experiences. They found it helpful to be able to add detail and a narrative around the negative moments to give them context. They spoke about the significance of being able to see negative memories alongside other memories and realise how these negative memories could be contributing to low mood.

Like me opening up about my secondary school experiences, I felt like I was able to relieve such emotions and…deal with such negative thoughts. (Ruby)

Participants spoke about the importance of being able to strike a balance between not pushing away the bad memories but also not over-thinking the memories; describing that processing the memories allowed them to reduce intrusions and move forward from the experiences.

I remember at the time still having nightmares about it… [following the session] you think about it a bit more, which it allowed me to do, and it didn’t just take one day, I’m not going to lie and say it was a quick fix, but over a few months and like talking to my mum and getting things out in the open and [now] I don’t think about it at all, well obviously there’s the odd little twinge that comes up, but you’re able to deal with them. (Andy)

Some participants spoke about finding it challenging to remember these negative memories but valuable and important for therapeutic change. However, one participant said that they found remembering a negative image unhelpful as it negatively impacted on their mood. He reported that the impact on his mood lasted for a day or 2 and that he had not thought about the memory since (i.e., he has not experienced any intrusions from the memory). The remaining 11 participants spoke about using similar techniques to process other negative memories and to make changes, outside of the intervention. They reported being able to think through their past experiences more carefully, appreciate when their behaviour was unhelpful in real time, and act differently.

Even though it was all about memories and remembering the past, it’s also kind of like letting go of it and thinking towards what can be different...when me and my dad are in arguments now, I don’t like bring up what’s happened before…take a moment to calm down, then we think about it and then that’s like resolved the issue. (Alexandra)

*1.2.1 Compassionate perspective taking (n* = *9)* Participants identified that viewing the negative memory from an observer view, as a compassionate other, was helpful to see another person’s perspective and to work out what they would say in this situation. This technique helped them to think about other ways to approach situations and they identified that sometimes they were deriving meanings from experiences that were not necessarily accurate. However, one participant said that they had found trying to remember the memory from another perspective (their brother’s) uncomfortable (“*cringy”*). On elaboration, it appeared that this was because they did not have a compassionate relationship, with the participant saying that it would have been better if he had chosen his father or mother. Participants also gave examples of applying this strategy to other memories and to their everyday lives:

See it from the perspective of my parents…helps me to understand that I’m putting too much pressure on myself …I need to think about myself first, and that my parents are probably not going to be too upset with me if I fail…it helps me to calm down. (Eleanor)

**1.3 Imagining positive future events** Eleven participants highlighted that creating detailed future images had been important for change. Generating positive future images had generated positive emotions, such as excitement, and increased their motivation.

Imagining myself in that position was really eye-opening and…now I’m motivated to work harder to see myself in that position (Mishti)

Participants reported that imaging the future had enabled them to structure their future and formulate how they would achieve their goals. Participants spoke about how the exercise and being able to visualise their end goal helped them to set more intermediate goals and the methods to achieve these.

What you need to do in order to achieve that, how you can achieve those goals, it just depends on what you do right now and the steps you take. (Ruby)

They now imagine a more positive future, whereas previously negative future imagery dominated. The detail that they generated the images in was important to participants, with them identifying that this increased their ability to imagine the scene which increased their motivation.

Yeah, because you would like describe the colours of the walls and how all the kids are, and yeah I can really picture this…it motivated me more to want to get there. (Prisha)

Participants identified that they now had a new appreciation of the importance of being able to be resilient and flexible when plans change, or goals are not achievable.

There’s every stepping stone, and every stepping stone one of them could be loose and you could go back one, so it was not just about looking at the future, it was about looking at and making your way through…it’s how you deal with the step backwards that leads to you going forwards. (Andy)

Interestingly, some identified that creating a future image helped them to appreciate that there are multiple things they might want to do in the future and to think about different aspirations (e.g., one participant spoke about being very focussed on becoming a police detective and that the exercise had helped her to appreciate that she has lots of other skills that she also wants to pursue). Participants gave examples of having used this technique to generate other positive future images, such as future holidays, alternative career options, and achievements at school.

**1.4 Understanding and being kinder to myself** This theme reflected the sense that participants gave of being able to have a more complete understanding of themselves and how their thoughts, feelings, and behaviours operate. They identified that this allowed them to be more compassionate to themselves. For example, participants described a sense of being more aware of their negative thoughts and being able to challenge them compassionately. There were three subthemes:

*1.4.1 Expressing emotions and understanding myself (n* = *8)* Participants spoke about the importance of having the opportunity to talk to someone confidentially and the time to be able to go into detail about their lives in a way they had not had the opportunity to do before. They highlighted that expressing emotion brought a sense of relief. The intervention had improved their understanding of themselves and enabled them to make changes to improve their mood and seek support from others. During the intervention, memories are imagined in detail and grouped together in categories; participants identified that having clearer memories allowed them to express themselves more.

Before…I don’t have the clear memories, I feel like I can’t express myself, like I don't know, like it’s blurred, I don't know what words to use or what words would be right. Because of the groupings, of when we group the words, and then the clear memory, I can express myself more. (Suravi)

*1.4.2 Self-compassion (n* = *6)* Changing their attitude to be kinder to themselves has been significant to participants and had helped them cope with low mood. They identified that the intervention had been useful to reflect on how they were currently feeling and prioritise their own needs.

It helped me think more about myself as an individual and how I'm as important as each other person, because I do tend to do that a lot, I focus more on how other people are feeling and how other people are doing rather than myself. (Sara)

*1.4.3 Values for living (and links with memories) (n* = *10). *Thinking about what is important to them and seeing their memories in the context of their values had been helpful to improve mood. Participants spoke about the exercise broadening their understanding of themselves and being able to see the different aspects that influence them.

I was quite focused on just like getting the right grades and I really didn’t take the time about all the other things that are going on around me and like the relationship with your parents and your friends and being with your friends…it [thinking about my values] did kind of like take away from much of my stress and anxiety. (Mishti)

They also identified that it had been interesting to think about (after the session) the similarities and differences they share with their friends and what they would like to seek out in future friendships.

2. **Outcomes of the intervention**

Six themes emerged within the category of outcomes of the intervention (please see Table 2 for a summary).

**2.1 Improving mood and well-being (n = 10)** Participants consistently identified that the intervention helped them to increase positive mood and reduce negative emotions. They reported being able to appreciate both the negative and positive aspects of experiences, rather than focus only on the negative. They identified being able to feel calmer, think through their experiences more, and be more open-minded.

I feel less guilty, I don’t know why but I used to feel really guilty…I just feel like just being able to not think so much about negative things, makes me happier, like I’m able to just enjoy myself. (Prisha)

**2.2 Reducing impact of negative memories (n = 11)** A major outcome of the intervention for participants was to think about their negative memories differently and to diminish the power of these memories.

It wasn’t like a healthy way of remembering things basically. [Now] I think about what actually happened that’s making me upset and obviously that’s how I can progress in the future, thinking about, like, OK, this is what needs to be different. (Alexandra)

Participants described being able to transform the negative memories from something that they found very upsetting and often thought about to something that they were no longer bothered by. One participant even described now finding the memory “*funn*y”.

*2.2.1 Overcoming rumination to the memory (n* = *3). *Participants spoke about no longer repeatedly thinking about the memory. They spoke about how this rumination had previously affected their mood, but now they were able to see these memories as events in their lives rather than something that defined them.

[Bullying is] something that can really affect you mentally, physically, and not just like for the moment that it’s going on, it can affect your whole life. It was constantly just something that I was worrying about…And so for me to just stop worrying about that, it was good, I'm more careless about it now…I'm more likely to stand up for myself. (Sara)

**2.3 Motivation and goal-directed behaviour (n = 10)** A key recurrent theme was that the intervention improved participants’ ability to motivate themselves to achieve their goals. Participants spoke about how creating detailed positive future imagery enabled them to identify future goals, make the goals feel more achievable, and highlight the intermediate steps to reach these goals.

Do those little steps for that, before that big step… I’m a bit more organised with like my subjects and what I do, and I know I’m a bit more know what I’m doing, like I don’t really miss stuff as much as I used to, or at all now, I’m just kind of on it and I do it. But I feel like a year ago I wouldn’t really be able to do that. (Ben)

Participants also spoke about previously feeling disheartened when they did not achieve a smaller goal, whereas now they were able to put this in context and so it did not impact on their mood and behaviour as much. This enabled them to continue working towards their goals and remind themselves of other occasions when they had achieved goals.

**2.4 Overcoming avoidance and rumination (n = 6)** Participants described a sense of being able to activate their behaviour rather than being consumed by negative thinking cycles. They spoke about being able to process negative emotions and be able to quickly move on from them. Participants gave examples of being able to engage in activities (especially with friends), which previously they would have avoided.

[The intervention] took away a little bit of the insecurities that I had… Yeah, accept myself so I could then talk to people.... because I over-think, like people who hate me, well, they don't always think that, I'm just assuming that they do… like you helped me talk to people without thinking what they, if they judge me or not, and for how they judge me, I stopped thinking about that, I just talked to them. (Tanay)

**2.5 Relationships, communication, and being open (n = 12)** Being able to express themselves more following the intervention and allowing themselves to show their vulnerabilities to others was important to participants. They gave examples of being able to approach people and speak to them in ways that they would not have done previously; this included being more confident to make new friends, speaking to parents, and speaking about their emotions with friends.

I kind of opened up, decided to talk to the teachers more, and yeah, doing that helped me. (Michael)

Participants identified worrying about being judged by others or not standing up for themselves, whereas now they felt able to approach people and be confident.

I’m allowing more people in now, like my best friend, never been around my house… now he’s like round most of the time playing Xbox and we’re just talking and having fun because it’s just because of like me not contemplating on stuff that I didn’t want to contemplate on and not letting people see [my family] (Andy)

**2.6 Self-understanding and acceptance (n = 12)** Participants referred to feeling an increased sense of being able to accept themselves for who they are and that this had led to improved confidence and enabled them to approach situations differently. They spoke about being much more aware of how their negative thoughts might be influencing their feelings and behaviour and that these thoughts were not based on any evidence. Alongside this was the sense that negative thoughts about themselves no longer dominated their thinking and they were more equipped to balance this with positive thinking.

Well I used to have insecurities about coming to college and seeing everyone…made me realise that I’m not actually like a bad person, it made me realise that I am good and that I am caring, and all of the fears that I used to have, and all of the negative thoughts that I used to have, they all kind of subsided... sometimes they come back, but I know how to handle it now and to stop it from actually taking over my life. (Eleanor)

3. Integration of intervention components, active ingredients, and outcomes

**Fig. 1:**
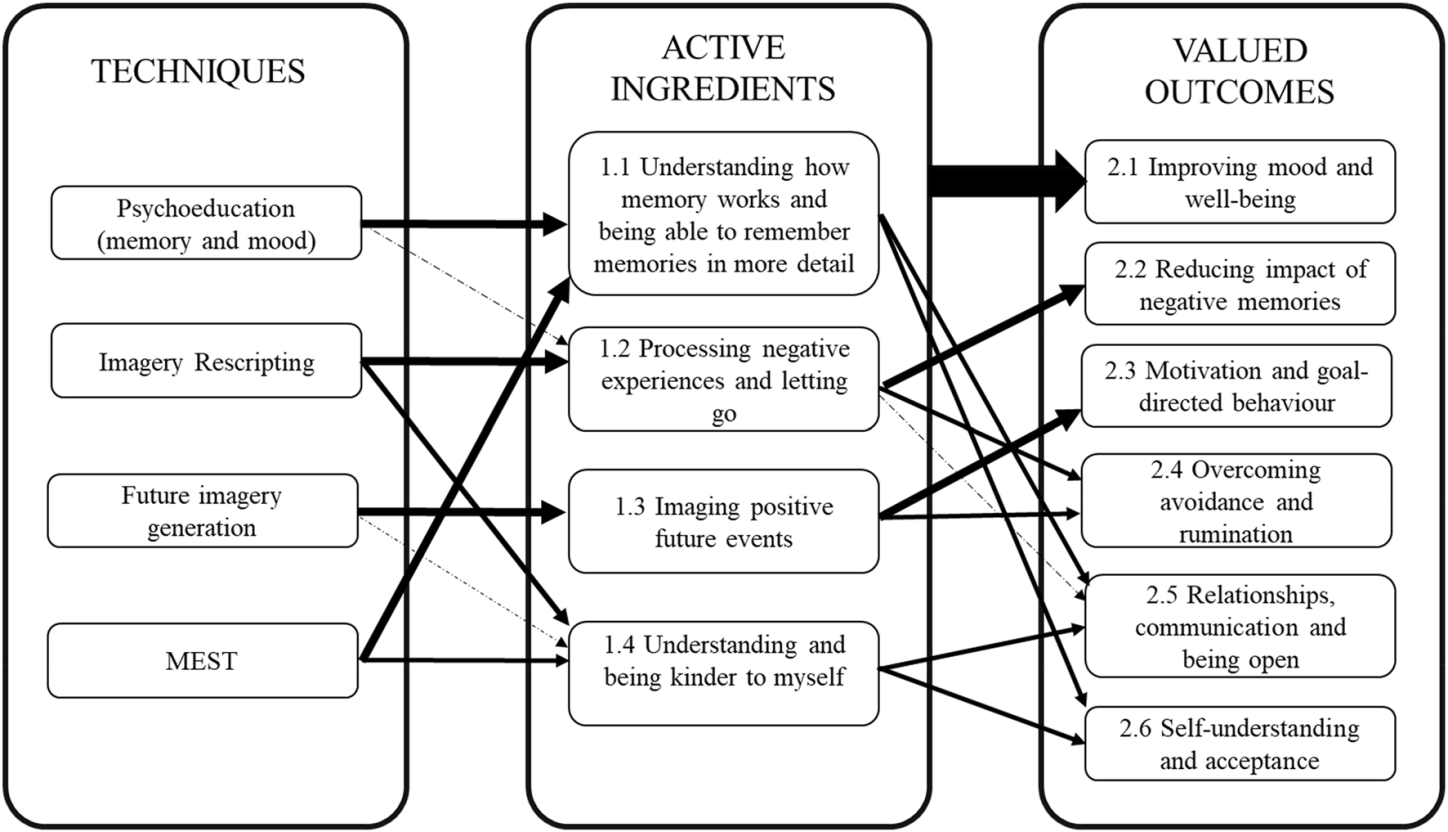
Simplified logic model integrating the intervention components, active ingredients, and valued outcomes

Please see Fig. 1 for a simplified logic model. The four different intervention components appeared to be associated with their complementary active ingredients (e.g., IR associated with 1.2, as described in the above quotes; for example, one participant spoke about “letting go of it [the memory]” following IR*)*. However, psychoeducation and MEST were linked most strongly to one active ingredient (1.1), and 1.4 was associated with a number of the intervention components.

In terms of valued outcomes, all the active ingredients were discussed in terms of generally improving mood and well-being. There were then clear links between 1.2 and 2.2 as well as 1.3 and 2.3. Ingredients 1.2 and 1.3 also contributed to 2.4, and not only specifically to memories but rather avoidance and rumination more generally (e.g., “it’s how you deal with the step backwards that leads to you going forwards”). 1.1 and 1.4 were associated with both 2.5 and 2.6. Young people included an element of improved self-understanding in 1.1 and 1.4. Young people spoke about improved self-understanding in combination with the “evidence” from having detailed memories (1.1), as being helpful for self-acceptance and communication with others.

## Discussion

The findings suggest that some of the active ingredients were similar to what we expected, justifying the theoretical basis of the intervention. They also identify additional ingredients, extending our understanding of how the intervention might work. Participants were able to identify multiple outcomes resulting from the intervention that they valued. The findings are an important contribution to our understanding of how ICBI (and psychological interventions more generally) might work to improve depression in adolescence. They provide a rich insight as to how participants interacted with the key techniques and what they derived from them. It also underlines the value of using mixed-methods approaches in the evaluation of complex interventions, enabling the detailed exploration of participant views on the mechanisms of action and the ways in which the intervention could be improved.

Promisingly, participants clearly articulated what they found helpful about the intervention and the changes that they had experienced as a result of the intervention. In many respects, their subjective accounts of the active ingredients confirmed the views held by the psychologists who developed the intervention. Given that the intervention has multiple components coupled with the complexity of the depression presentation, it is reassuring that the participants were so consistent in identifying what they found helpful about the intervention. Processing negative experiences and imaging positive future events emerged as important aspects of the intervention to drive change. An important element of processing negative experiences that emerged was the ability to let go of these experiences. One participant did identify not finding the negative image rescript helpful. It would be useful to understand whether there are factors at baseline that could predict which participants would benefit from the negative image rescript and which would not. In the analysis, understanding how memory works and being able to remember memories in more detail was grouped as one theme to reflect how this was communicated by participants in the interviews. Previously, we had considered this to be two separable ingredients. It was interesting that understanding and being kinder to myself emerged as a principal theme as although this is an element of the negative image rescript (where the participant views the image from the perspective of a compassionate other), it is not a primary focus of the intervention. Developing a clear understanding of the likely active therapeutic ingredients is helpful to enable the intervention to be fine-tuned but also to ensure that these are retained when the intervention is scaled up and/or delivered by practitioners with varying backgrounds and experience. This increases the likelihood that the large effect sizes that were achieved in trial are replicated. However, it should be noted that the trial therapist conducted the interviews, and this may have introduced bias in which ingredients were highlighted. This decision was made to maximise recall of the intervention content and open questioning was employed throughout the interview. Future trials would perhaps benefit from interviewers who have delivered the intervention previously (and so are familiar with it), but are independent from the interviewee.

Similarly, the themes for the outcomes that were valued by participants were partly in keeping with what we might have predicted but also new understandings emerged from the data. For example, the key aims of the intervention included improving mood and well-being and reducing impact of negative memories. Increased motivation and goal-directed behaviour were an outcome that we hoped would result from the positive image rescript (and increased access to positive experience), but these techniques are very novel. It is encouraging that participants clearly identified that these three themes/outcomes (a) resulted from the intervention and (b) were important to them.

The other three themes were less predictable from our knowledge of the intervention. Overcoming avoidance and rumination (generally, rather than to memories specifically) is promising given that approach behaviours are linked to recovery from depression and targeted in more traditional psychological approaches to depression [32–34]. A key element often identified in recovery is improved social connection [35, 36] and a major focus of relapse prevention is being able to seek help, so it is positive that young people felt that the intervention had improved their relationships, communication, and ability to be open. Similarly, self-acceptance and understanding (i.e., self-compassion) have been suggested as a resilience factor that protects against the development and maintenance of depressive episodes [37]. Interestingly, research suggests that being able to tolerate negative emotions is an important mediating factor in the relationship between depression and self-compassion [38], and a key message for the negative imagery rescript is to be able to tolerate the associated negative emotions. What we cannot know is whether these outcomes are specific to this intervention or whether they would have also been identified by young people in the control group (who received non-directive supportive therapy). Future research could compare those receiving different types of intervention to see whether there is specificity in the emergent themes, as well as investigate whether those who experience a greater reduction in depression symptoms report different types of outcomes compared to those who show less reduction. Furthermore, we did not draw any conclusions about the links between gender and/or ethnicity and the active ingredients and valued outcomes. There was a relatively small sample within the different subgroups and no clear differences from the data between the subgroups. Future research with larger samples could explore whether the results vary for different genders and/or ethnicities.

Figure 1 aims to link the intervention components to the active ingredients and valued outcomes. Our findings are consistent with previous qualitative research that has helped to elucidate young peoples’ understanding of why they are depressed [39]. For example, themes such as “depression as a result of rejection, victimisation and stress” are linked to 1.2 (processing negative experiences) and to 2.2 (reducing the impact of these negative memories). Similarly, “bewilderment about why they were depressed” and “something inside me is to blame” appear to be linked to 1.1 (understanding how memory works) and 1.4 (understanding and being kinder to myself), 2.4 (reducing avoidance and rumination), and 2.6 (self-understanding and acceptance). Furthermore, research on the experience of depression [29] highlights themes such as “a bleak view of everything” and “isolation of cutting off from the world” which links to 1.3 (imagining positive future events) as well as 2.3 (motivation and goal-directed behaviour) and 2.4 (overcoming avoidance and rumination). Together, these suggest that the active ingredients are targeting factors that adolescents identify as important in their depression and that the intervention is resulting in valued outcomes that are incompatible with their experience of depression (and thereby reducing it).

Our findings highlight the benefits of utilising mixed-method approaches for evaluating complex interventions in RCTs. A sole focus on the quantitative outcomes would not have enabled us to identify the mechanisms through which the intervention led to therapeutic change. This study supports the MRC guidance on process evaluation as a useful component of evaluating complex interventions [17]. Whilst co-design and co-production with service-user consultants were key in this research and in the RCT more generally, we did not formally evaluate this process. Although co-production is recognised as a highly valuable process [40], it lacks extensive evaluation to enable evidence-based recommendations for meaningful co-production. Previous research [40, 41] has made recommendations (such multi-stepped participatory approaches) which could usefully be incorporated, adapted, and evaluated in future research.

The findings here improve our understanding of which intervention components are actively contributing to change and, therefore, are core components and should be retained in the intervention and studied in future research. More generally, this study suggests mechanisms by which techniques for adolescent depression may work and which elements are important. For example, IR is often incorporated into cognitive behavioural therapy for adults and this work suggests that it is likely to also be helpful to youths with depression. It is important to note how the changes in depression scores (the primary clinical outcome) may or may not accurately reflect the experience that participants describe having in the intervention. For example, Michael spoke about finding IR unhelpful yet showed a decrease in his depression score that was clinically meaningful at T2, whilst Prisha spoke about finding the intervention helpful and yet showed an increase in her scores at T3. This discrepancy highlights the importance of combining both qualitative and quantitative techniques to understand meaningful change for a given individual both in research and in clinical practise. In addition, future research could consider more innovative multi-modal methods to engage participants in the collection of data (e.g., photoelicitation) and co-production of data collection methods (e.g., photovoice) [42, 43].

This study did have several limitations. One limitation is that we were unable to interview the one participant who dropped out during the intervention to understand whether this was due to a particular feature of the intervention. Further to this, three participants did not reply to the invitation to take part. This means that the full range of perspectives and experiences of the study may not have been included. A further limitation for the study is that it is not possible to estimate the relative benefits of each ingredient. According to implementation fidelity theories [44], it may be important for future studies to separate the “hard-core” and “soft periphery” components to enable meaningful implementation of the intervention across settings or countries [45, 46]. Perhaps unsurprisingly, it appeared that some components were particularly valuable to some participants, whilst other participants more strongly endorsed other factors. What we cannot know is whether we could have predicted this based on baseline characteristics and so tailored the intervention to individuals to maximise effectiveness. For example, it could be that the negative image rescript is particularly beneficial for young people struggling with intrusive images of the past. Tailoring the intervention would be useful to explore in future research. There is potential for our findings to be affected by recall bias as participants were interviewed about the intervention that they had completed 3 months previously. We attempted to counteract this by the trial therapist conducting the interviews and using visual cues in the intervention workbook. However, this also introduced the limitation that there was a lack of independence between the intervention and interviews as this may have introduced demand effects. Whilst the interviewer/therapist was highly conscious of asking any leading questions or prompts which might result in bias, this could have meant that participants were less likely to provide constructive criticism and/or may have remembered the intervention differently if interviewed by someone independent. However, constructive criticism was generated; for example, one participant identifying IR as unhelpful, and another finding the perspective taking element of IR uncomfortable. There were also unexpected insights into the mechanisms; for example, one participant identified being able to think more broadly about her future aspirations. On reflection, VP was highly invested in the intervention but also viewed this stage as a key developmental phase for IMAGINE (before testing in a larger trial) and so eager to hear constructive feedback that could maximise its potential. A principal reflection that VP made in supervision was that greater insights were garnered from this study than expected, partly driven by participants being more psychologically minded and remembering more of the intervention than predicted.

Our findings indicate that an intervention for adolescent depression, designed to improve access to treatment in resource-constrained settings, has a justified theoretical framework. The next stage for this promising intervention is to scale it up and for it to be delivered by practitioners without extensive training. Identifying the active ingredients and the values outcomes will augment the ease of achieving this and our understanding of how to replicate the effects.
